# Data for iTRAQ-based quantification of the effect of HuganQingzhi on non-alcoholic fatty liver disease in rats

**DOI:** 10.1016/j.dib.2017.10.027

**Published:** 2017-10-16

**Authors:** Xiaorui Yao, Fan Xia, Waijiao Tang, Chunxin Xiao, Miaoting Yang, Benjie Zhou

**Affiliations:** a Department of Pharmacy, Shantou Central Hospital, Affiliated Shantou Hospital of Sun Yat-Sen University, Shantou 515041, Guangdong, PR China; b Department of Pharmacy, The Seventh Affiliated Hospital, Sun Yat-sen University, Shenzhen 518107, Guangdong, PR China; c Center for Drug Research and Development, Zhujiang Hospital, Southern Medical University, Guangzhou 510282, Guangdong, PR China

## Abstract

The data presented in this article are related to the research article entitled “Isobarictags for relative and absolute quantitation (iTRAQ) -based proteomics for the investigation of the effect of HuganQingzhi on non-alcoholic fatty liver disease in rats” (Yao et al., 2017) [1]. This article describes the effect of HuganQingzhi on non-alcoholic fatty liver disease in rats at the level of the proteome (HFD: control, HH: control, HH: HFD, respectively). The field dataset is available to criticize or extended analyzes in public.

**Specifications Table**

**Table:** 

Subject area	*Pharmacology*
More specific subject area	*Ethnopharmacology*
Type of data	*Tables, Text file*
How data was acquired	iTRAQ*, LC-MS/MS*
Data format	*Globally normalized quantitation and analysis*
Experimental factors	*Protein was extracted from each group (control, HFD and HH) and quantified with iTRAQ*
Experimental features	*The difference of protein expression level among three groups (control, HFD and HH)*
Data source location	*Guangzhou, China*
Data accessibility	*The Data are available with this article*

**Value of the data**

1. The data make us better known about the pathogenesis of NAFLD and could be used by other researchers for further study.
2. It is an urgent need to further investigate the proteins expression changes which are associated with the treatment of HQT in HFD-induced NAFLD rats.
3. The data provide deeper insight into many cellular pathways and elucidate the underlying mechanism of the effects of HQT in NAFLD treatment.

## 1 Data

The original MS/MS file data were performed using the Paragon algorithm [2] as implemented in ProteinPilot Software v4.5. Supplemtary Table 1 lists the data of protein identification and quantification including Uniport Accession, Protein description, Protein unused, Protein mass, Total peptide matches, Sequence Coverages (95%), Unique Peptide Sequence and average fold change of each pair. Supplemtary Table 2 shows the differentially expressed proteins (DEPs) identified by iTRAQ analysis in HFD: control, HH: control, and HH: HFD, respectively. Supplemtary Table 3 lists the pathways, which are annotated in HFD: control, HH: control, and HH: HFD, respectively.

## 2 Experimental design, materials and methods

**Fig. 1:**
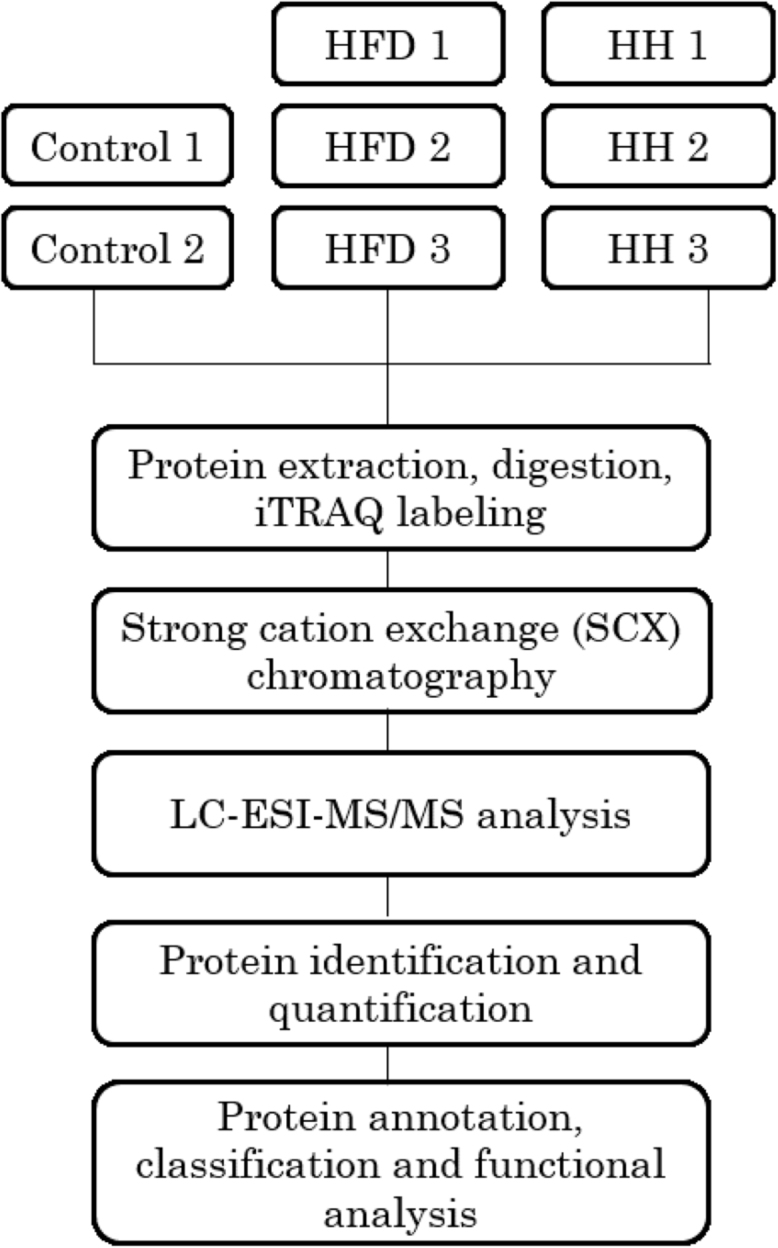
Flow chart of data on iTRAQ experiments and subsequent bioinformatics analysis.

Full methodological details are available in [1]. For data on iTRAQ experiments and subsequent bioinformatics analysis, a flow chart related to the associated research article was shown in Fig. 1.

### 2.1 Research animals and experimental design

All procedures are described in the associated research article [1].

### 2.2 Protein extraction and digestion and labeling

All procedures are described in the associated research article [1].

### 2.3 Strong cation exchange (SCX) chromatography

All procedures are described in the associated research article [1].

### 2.4 Nano-liquid chromatography and mass spectrometry (MS) analysis and data analysis

All procedures are described in the associated research article [1].
